# Photoactive Hydrogel-Based Therapy for Biofilm Disruption in Chronic Wound Infections

**DOI:** 10.1021/acsomega.5c02315

**Published:** 2025-07-11

**Authors:** Wanessa CMA Melo, Eglė Žalytė, Adei Abouhagger, Gabija Lauciu̅tė, Antanas Straksys, Arunas Stirke

**Affiliations:** 226274 State Research Institute Center for Physical Sciences and Technology (FTMC), Department of Functional Materials and Electronics, Vilnius 01133, Lithuania

## Abstract

This study presents a novel approach for treating chronic biofilm-associated infections by utilizing a gel-like hydrogel (HG1MB1) incorporating methylene blue (MB), a photosensitizer, to enhance photoinactivation of microbial biofilms. The key novelty of this work lies in the gel-like structure of HG1MB1, which significantly improves the penetration of MB into the deeper layers of the biofilm, promoting more effective disruption of the biofilm matrix. This improved uptake and interaction with the extracellular polymeric substances (EPS) of the biofilm leads to a reduction in EPS and breakdown of the biofilm structure. Additionally, the hydrogel’s ability to generate reactive oxygen species (ROS) upon light activation enhances its antimicrobial efficacy. HG1MB1 also demonstrated broad-spectrum activity against both bacterial and fungal biofilms, suggesting its potential as an effective treatment for chronic infections.

## Graphical abstract

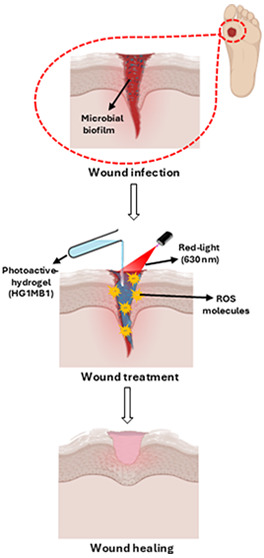

## Introduction

Chronic wound infections pose a major healthcare challenge, often leading to delayed healing, increased morbidity, and higher risks of severe complications such as sepsis and limb amputation.^1,2^ The persistence of these infections is largely attributed to microbial biofilms, which enhance antimicrobial resistance and immune evasion.^3,4^ Among the most problematic pathogens, *Pseudomonas aeruginosa*, *Staphylococcus aureus*, and *Candida albicans* play a crucial role due to their virulence, adaptability, and ability to form complex microbial interactions.^1^ *P. aeruginosa* is frequently found in burn wounds and chronic ulcers, exhibiting strong biofilm formation and multidrug resistance.^2^ Meanwhile, *C. albicans*, though a commensal fungus, becomes pathogenic in immunocompromised patients, exacerbating bacterial biofilm formation and infection severity. *S. aureus*, the most common cause of wound infections, contributes to chronic wound persistence through its ability to form biofilms and produce a range of virulence factors that promote tissue damage, immune evasion, and sustained infection.^3^

Hydrogels have emerged as a promising strategy for chronic wound management due to their ability to maintain a moist wound environment, promote tissue regeneration, and serve as carriers for antimicrobial agents, nanoparticles, and bioactive molecules.^5^ Functionalized hydrogels incorporating silver nanoparticles, antimicrobial peptides, or quorum-sensing inhibitors have demonstrated potential in biofilm disruption and microbial load reduction.^4^ However, conventional hydrogels face limitations such as insufficient biofilm penetration, short-lived antimicrobial activity, and potential microbial adaptation. Some formulations may also induce cytotoxicity or inflammatory responses, raising concerns about their long-term safety and efficacy.^5^

To overcome these challenges, photoactive hydrogels combined with antimicrobial photodynamic therapy (aPDT) offer a novel and effective approach. aPDT relies on light-activated photosensitizers to generate reactive oxygen species (ROS), which induce oxidative damage to microbial cells, including multidrug-resistant bacteria and fungi.^6^ When incorporated into hydrogels, photoactive molecules such as methylene blue provide targeted, controlled antimicrobial action, minimizing off-target effects and reducing reliance on systemic antimicrobials.^5,7^ This strategy significantly enhances biofilm penetration, broad-spectrum antimicrobial activity, and wound healing outcomes.^7^

Given the growing challenge of multidrug-resistant infections, integrating photoactive hydrogels with aPDT presents a cutting-edge, multifunctional therapeutic approach for chronic wound management.^4^ This study investigated the photoactive properties of a gel-like methylene blue-loaded hydrogel as a strategy to combat biofilms formed by *C. albicans, P. aeruginosa, and S. aureus*. Additionally, the study evaluates the cytocompatibility of the hydrogel with skin fibroblast cells, ensuring its potential for safe and effective application in chronic wound treatment.

Additionally, fibroblasts play a central role in the dermal layer of skin and are crucial for wound healing, extracellular matrix remodeling, and tissue repair processes. In the context of chronic wounds, fibroblast activity is often impaired due to infection and inflammation. Assessing the impact of the photoactive hydrogel on fibroblasts therefore provides relevant insight into its potential for promoting tissue regeneration and healing. While keratinocytes are also important in re-epithelialization, fibroblasts serve as an initial indicator of cytotoxicity in the deeper layers of the wound bed, which are more likely to be exposed to the hydrogel during clinical application.

Thus, by utilizing light activation to generate ROS, this approach aims to enhance antimicrobial efficacy while minimizing the limitations of conventional hydrogels.

## Results

### Uptake of Methylene Blue from Photoactive Hydrogel (HG1MB1) by Microbial Biofilm and Fibroblast Cells

The uptake of MB in both aqueous solution (MB1) and hydrogel formulation (HG1MB1) is presented in Figure 1. Overall, MB binding was significantly higher in biofilms than in fibroblast cells, which reached only ∼ 20% uptake after 120 min. In contrast, biofilms achieved this level much faster (within 20 min for MB1 and just 5 min for HG1MB1), indicating a strong selective effect. This pattern remained consistent throughout the experiment.

**Figure 1:**
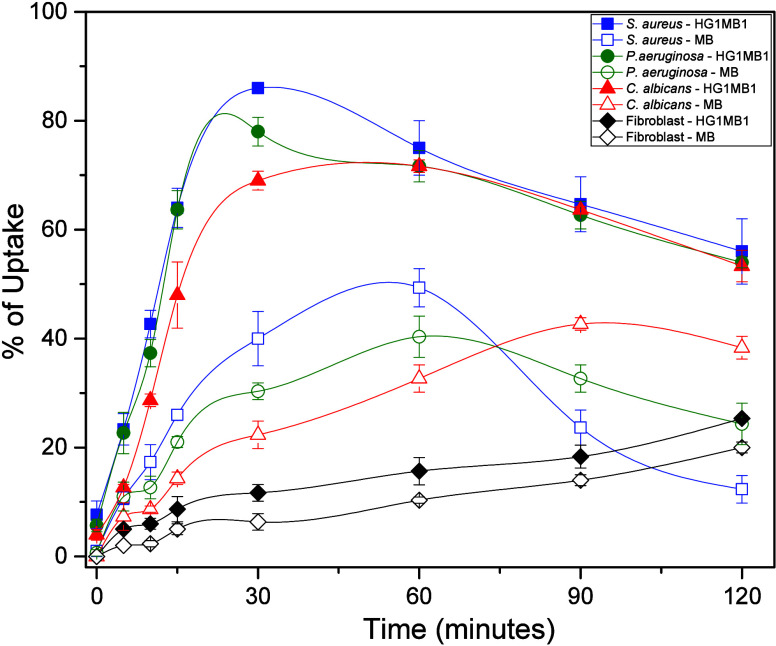
Uptake percentages of MB in both aqueous solution (MB1) and hydrogel formulation (HG1MB1) by biofilms of *S. aureus*, *P. aeruginosa*, *C. albicans*, and Fibroblast cells. Values are means of three replicates and bars are standard deviations.

It is important to highlight that dye uptake was both faster and higher with HG1MB1, particularly in biofilms, reaching maximum levels about 30 min earlier than with MB1. The highest uptake values for HG1MB1 were 86% for *S. aureus*, 78% for *P. aeruginosa*, and 71% for *C. albicans*, whereas MB1 uptake reached only 49%, 42%, and 40%, respectively. Additionally, the differences in microbial morphology played a crucial role in uptake variations. *C. albicans* reached its maximum uptake level at 60 min, whereas *P. aeruginosa* and *S. aureus* achieved their peaks at 30 min. Among the microorganisms, *S. aureus* exhibited the highest MB uptake, followed by *P. aeruginosa*, with *C. albicans* showing the lowest maximum uptake level.

### aPDT Biofilm Inactivation by HG1MB1

The *in vitro* aPDT potential of the HG1MB1 against microbial biofilms and its effect on Fibroblast cells is presented in Figure 2. After 30 min of incubation at 37 °C and exposure to a light dose of 24 J/cm^2^, the hydrogel significantly (*p* < 0.0001) inhibited biofilms compared to all control groups (Table 1). Under these conditions, the viability of *S. aureus*, *P. aeruginosa*, and *C. albicans* decreased by approximately 5.42, 4.97, and 3.42 log_10_, respectively (Figure 2A). This represents more than a 2-fold reduction in comparison to the inhibition achieved by MB1 under the same conditions, where *S. aureus* viability decreased by 2.59, *P. aeruginosa* by 1.26, and *C. albicans* by 0.67 log_10_ (Figure 2A). These findings suggest that the hydrogel promoted a stronger effect against the biofilm, significantly reducing (*p* < 0.001) the total number of viable cells compared to MB1.

**Figure 2:**
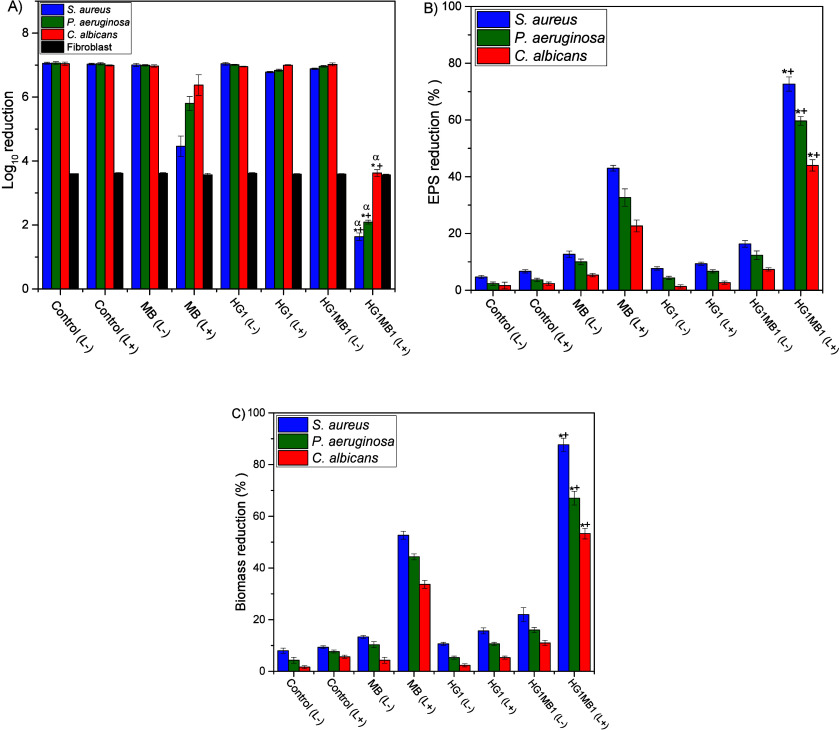
(A) Log reduction of microbial biofilms and fibroblasts after aPDT. (B) Percentage of biofilms-EPS reduction after aPDT. (C) Percentage of biofilm-biomass reduction after aPDT. Nonirradiated and irradiated cells are denoted as (L−) and (L+), respectively. Values are means of six replicates and bars are standard deviations. *p < 0.0001 versus its respective Control (L−); ^+^ *p* < 0.001 versus its respective dark control (L−) and MB1 (L+); ^α^ *p* < 0.005 versus fibroblast cells.

**Table 1:** Parameters Applied to the Control Experiments^a^

Controls	HG1MB1	MB water solution (1 mg/mL)	Incubation time (minutes)	Irradiation time (minutes)
L–	No	No	0	0
L+	No	No	0	30
MB (L−)	No	Yes	30	0
MB (L+)	No	Yes	30	30
HG1 (L−)	No	No	30	0
HG1 (L+)	No	No	30	30
HG1MB1 (L−)	Yes	Yes	30	0

a L– indicates non-irradiated samples (dark conditions), and L+ indicates irradiated samples exposed to red light (630 nm, 30 min).

The photoactive effect of HG1MB1 is further demonstrated by the EPS concentration (Figure 2B), where the reduction percentage was significantly higher in biofilms incubated with the hydrogel. EPS was reduced by 72%, 59%, and 44% in *S. aureus*, *P. aeruginosa*, and *C. albicans* biofilms, respectively. Notably, these reductions were approximately double those observed with MB1 under the same conditions. These results are consistent with those shown in Figure 1, where the penetration and/or binding of MB to biofilm cells and the matrix enabled significant inhibition of microbial cells and reduction of EPS following irradiation. Additionally, the reduction in EPS likely exposed a greater number of microorganisms to the aPDT effect after irradiation.

The reduction in both microbial cell viability and EPS is substantiated by a significant decrease in overall biofilm biomass, as illustrated in Figure 2C. Following treatment with HG1MB1 and subsequent irradiation, biofilm biomass was markedly diminished, showing reductions of 88% for *S. aureus*, 67% for *P. aeruginosa*, and 54% for *C. albicans*. These results underscore the efficacy of HG1MB1 in disrupting established biofilms across both bacterial and fungal species. The strong correlation between biomass reduction, EPS degradation, and decreased cell viability suggests that the disruption of the biofilm matrix weakens the structural integrity of the biofilm, thereby enhancing susceptibility to photodynamic treatment and promoting microbial cell death.

It is important to note that aPDT had a lesser effect on *C. albicans* compared to the bacteria, although a significant reduction in yeast was still observed, particularly when HG1MB1 was applied. This difference is likely attributed to the larger size and more complex morphology of *C. albicans*, as well as its ability to mount an effective response against oxidative stress through various defense mechanisms targeting ROS generated by aPDT.

Furthermore, fibroblast cells were significantly unaffected by aPDT under all conditions applied, reinforcing the selective nature of the treatment.

The dark controls (MB (L−) and HG1MB1 (L−)), light control without MB, as well as the HG1 (L+ and L-), did not inactivate the microbial biofilm, showing almost the same number of viable cells as the initial control (L−).

### HG1MB1 aPDT Mechanism of Action

The percentage of protein leakage was measured to assess membrane integrity in both biofilm cells and fibroblasts following aPDT treatment (Figure 3A). The percentage of protein leakage was significantly higher in biofilms following aPDT, with HG1MB1 showing a significantly superior efficacy compared to MB1 (*p* < 0.001). In *S. aureus* biofilms, protein leakage increased from 12.43% with MB1 to 55.62% with HG1MB1. Similarly, *P. aeruginosa* biofilms showed an increase from 10.01% to 47.05%, while *C. albicans* biofilms exhibited a rise from 6.48% to 37.18% following the same treatments. Among the biofilm cells, *S. aureus* exhibited the highest protein leakage, indicating greater susceptibility to aPDT-induced membrane damage compared to *P. aeruginosa* and *C. albicans*. Additionally, fibroblast cells demonstrated significantly lower protein leakage compared to biofilm cells, presenting 10% max of leakage when applied HG1MB1 (*p* < 0.001).

**Figure 3:**
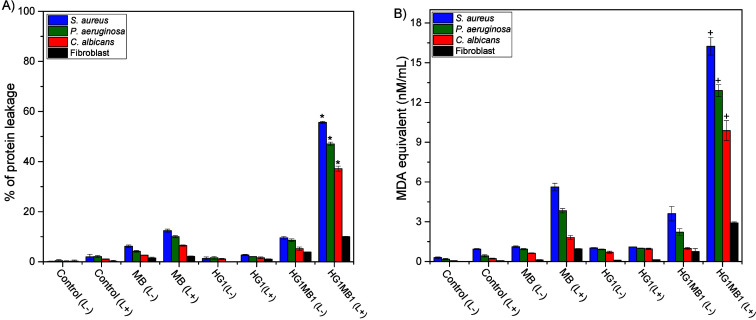
(A) Protein leakage and (B) lipid peroxidation from the microorganisms and Fibroblast after aPDT. Nonirradiated and irradiated cells are represented by (L−) and (L+). Values are means of six replicates and bars are standard deviations. *p < 0.001 versus its respective controls (all of them – Table 1) and fibroblasts HG1MB1 (L+). ^+^ *p* < 0.0001 versus its respective controls (all of them – Table 1) and fibroblast cells HG1MB1 (L+).

Lipid peroxidation in biofilm cells and fibroblasts was evaluated after aPDT with MB1 and HG1MB1 to assess oxidative damage, measured by MDA production (Figure 3B). The results aligned with protein leakage, as HG1MB1 was more effective than MB1 in inducing lipid peroxidation in biofilms after aPDT, with the highest effect observed in *S. aureus* (16.24 nM/mL). In the same conditions with HG1MB1, *P. aeruginosa* biofilms exhibited an MDA value of 12.90 nM/mL, while *C. albicans* biofilms 9.88 nM/mL.

Compared to biofilms, fibroblast cells exhibited 3–5 times lower lipid peroxidation, with MDA levels remaining below cytotoxic thresholds and at a nonlethal level. This highlights the potential of HG1MB1 for selective microbial inactivation with minimal damage to host cells. For both studies (Figure 3A and 3B), all dark controls (L−) and the control (L+) showed no significant results that would be considered impactful.

### Intracellular Measurement of ROS

The detection of total ROS in microbial biofilms and fibroblasts was evaluated using MB1 and HG1MB1, with DCF fluorescence intensity as an indicator after 30 min of irradiation at 630 nm (Figure 4). The results showed that after irradiation, HG1MB1 significantly enhanced ROS generation in microbial biofilms compared to MB1. Biofilms treated with MB1 exhibited a max fluorescence intensity of 9500 whereas HG1MB1-treated samples showed a markedly higher intensity of 12,383 (*p* < 0.001), indicating increased ROS production.

**Figure 4:**
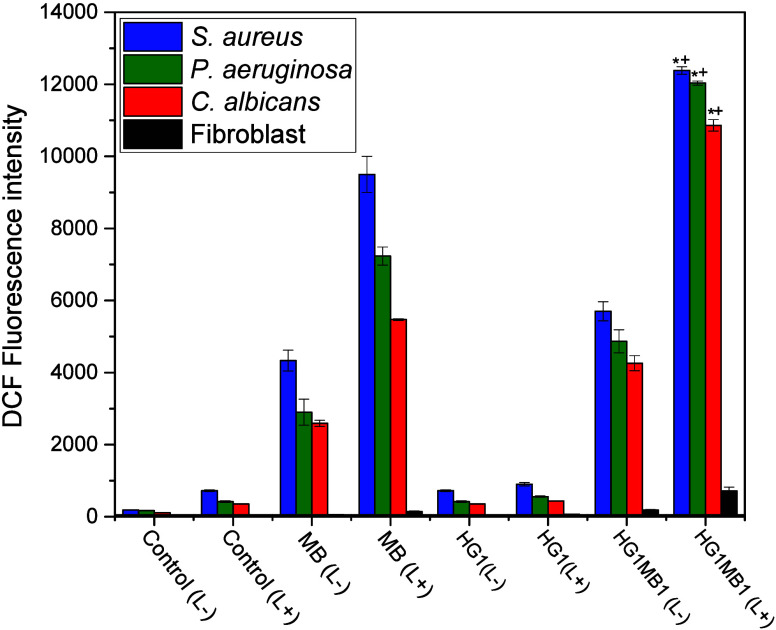
Detection of total ROS in the microorganisms and fibroblasts using MB1 and HG1MB1, monitored through the fluorescence intensity of DCF after 30 min of light irradiation at 630 nm. Nonirradiated and irradiated cells are denoted as (L−) and (L+), respectively. Values are means of three replicates and bars are standard deviations. *p < 0.001 versus its MB1­(L+) and its dark control HG1MB1 (L−) and ^+^ *p* < 0.0001 versus HG1MB1 (L+) in fibroblast cells.

Among the different microbial biofilms tested, *S. aureus* exhibited the highest intracellular ROS production, with a fluorescence intensity of 12,383, followed by *P. aeruginosa* (12,033) and *C. albicans* (10,862). However, these differences were not statistically significant (*p* > 0.05). This trend aligns with the overall enhanced ROS generation observed with HG1MB1, suggesting that while *S. aureus* may have a slightly greater intracellular ROS response, HG1MB1 effectively induces ROS production across all tested biofilms.

In contrast, fibroblasts did not show a significant increase in ROS levels after treatment with either MB1 or HG1MB1. The fluorescence intensity remained at 141 for MB1 and 716 for HG1MB1, suggesting that the hydrogel formulation did not enhance ROS generation in fibroblast cells under the tested conditions. This finding indicates that while HG1MB1 is highly effective at inducing ROS in microbial biofilms, its effect on fibroblasts is minimal, which could be advantageous for selective antimicrobial applications by reducing potential cytotoxic effects on host cells.

### Microscope Analysis of Fibroblast and Microbial Biofilms before and after aPDT with HG1MB1

The potential cytotoxic effects of HG1MB1 on HDFa were assessed through propidium iodide (PI) staining followed by fluorescence microscopy. The results demonstrated that HG1MB1 did not induce cytotoxicity under the experimental conditions, either in the presence or absence of irradiation (Figure 5). The treated fibroblasts retained membrane integrity, comparable to the control group. Moreover, PI staining did not indicate the presence of dead cells, as evidenced by the absence of red fluorescence signals in the microscopic analysis. These findings suggest that HG1MB1 does not compromise cell viability under the tested conditions.

**Figure 5:**
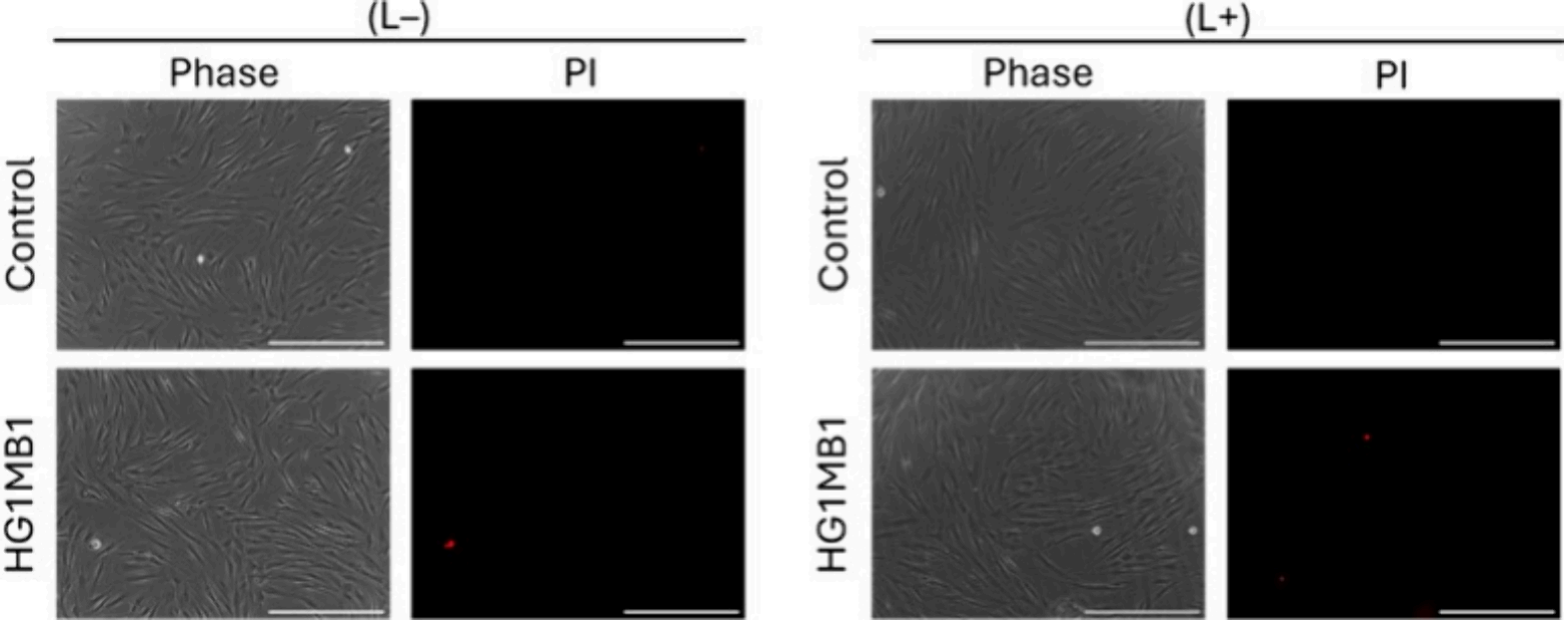
Representative phase contrast and fluorescence microscopy images of HDFa cells, untreated (control) and treated with HG1MB1. Nonirradiated and irradiated cells are denoted as (L−) and (L+), respectively. Cells were irradiated with light of 630 nm for 30 min. PI, propidium iodide. Scale bar – 400 μm.

In contrast, live/dead staining images revealed that biofilms treated with HG1MB1 exhibited increased red fluorescence (dead cells) and biofilm disruption compared to untreated controls, where biofilms remained largely intact with predominant green fluorescence (live cells), as shown in Figure 6. The extent of cell death varied among species, with *S. aureus* biofilms showing the highest reduction in viability.

**Figure 6:**
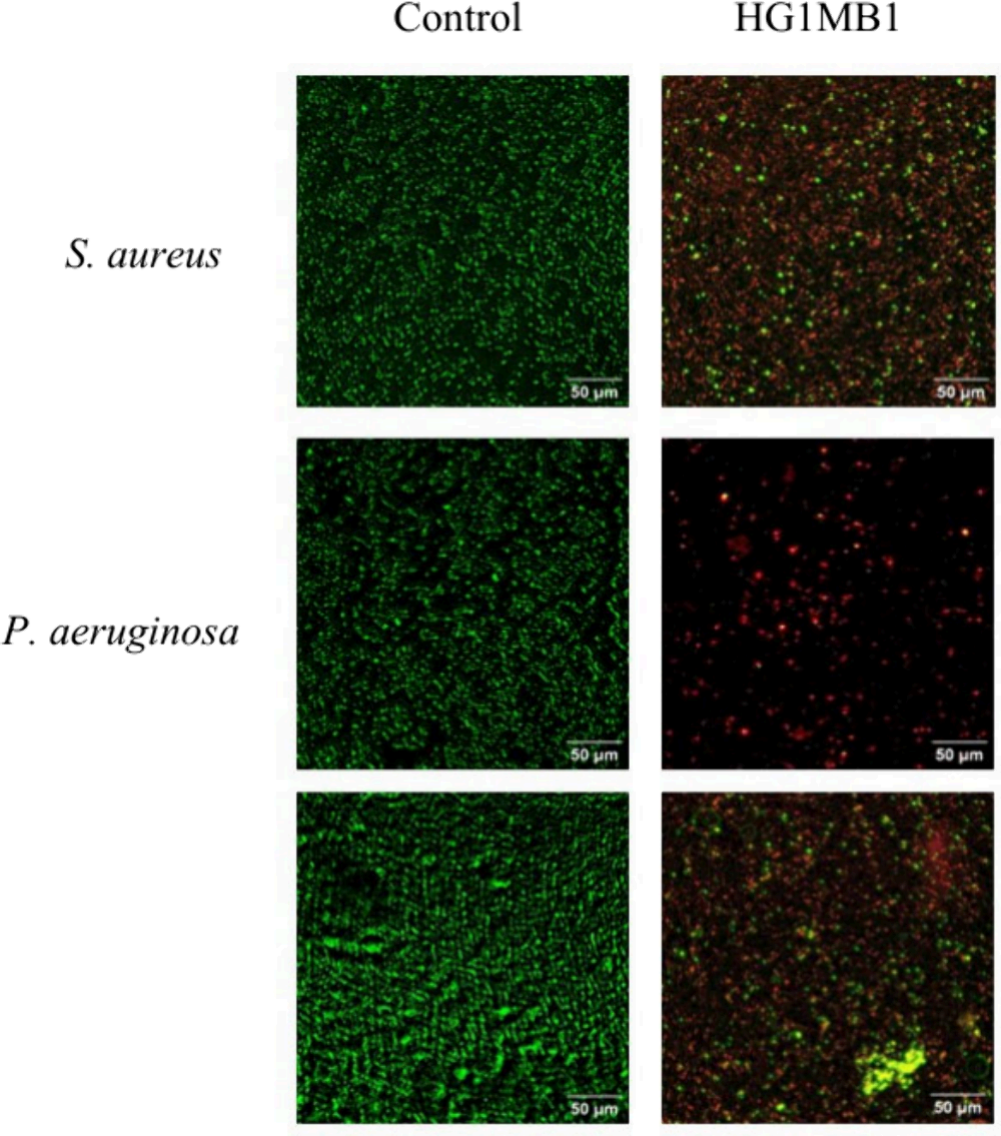
Live/dead staining of *S. aureus*, *P. aeruginosa*, and *C. albicans* biofilms using the MycoLight bacterial viability assay kit, comparing untreated controls to HG1MB1-treated samples. Green fluorescence denotes live cells, while red fluorescence indicates dead cells. HG1MB1 treatment led to enhanced cell death and biofilm disruption. Scale bars: 50 μm.

## Discussion

Chronic infections pose significant health challenges worldwide, often leading to prolonged illness, tissue damage, and diminished quality of life.^8^ These infections are typically caused by bacteria and fungi that resist conventional treatments, resulting in persistent symptoms and complications.^9^ The management of chronic infections is further complicated by factors such as antimicrobial resistance, inadequate immune response, and difficulties in reaching infected tissues.^8^ One promising solution emerging in recent years is the development of photoactive hydrogels, which offer unique advantages in treating these persistent infections. These hydrogels can be activated by light, enabling targeted and controlled release of antimicrobial agents at infection sites. This not only enhances the effectiveness of treatment but also minimizes side effects, making photoactive hydrogels a potential breakthrough in the management of chronic infections.^9–12^

Among the photoactive agents reported, MB is a widely recognized photosensitizer (PS) molecule, known for its relatively low toxicity and effectiveness against a broad range of Fungi, Gram-negative and Gram-positive bacteria. The high photoactivity of MB induces cellular damage, membrane lysis, and protein inactivation due to its high quantum yield of singlet oxygen generation. The primary objective of this work is to enhance the antimicrobial efficacy of MB dye by incorporating it into a hydrogel matrix.

This study assessed the effectiveness of a novel methylene blue-loaded hydrogel (HG1MB1) in targeting various microbial biofilms while minimizing damage to fibroblast cells, highlighting its potential for treating biofilm-related infections. Notably, HG1MB1 was selected for this study based on our previous research,^7^ which demonstrated its exceptional photochemical properties, including excellent thermal stability, predominantly elastic behavior, non-Newtonian shear-thinning characteristics, and efficient ROS generation upon light activation.

These characteristics of HG1MB1 played a central role in the breakdown of the biofilm matrix and the inactivation of microbial cells. Starting with its gel-like structure, which enhanced the uptake of MB within microbial biofilm since allowed the hydrogel to penetrate the biofilm matrix more effectively. This increased interaction facilitates more efficient dye diffusion through the EPS reaching the deeper layers of the biofilms. Similar observations have been reported by Zhang et al. (2016)^13^ and Shukla et al. (2020),^14^ who found that hydrogel-based systems improve antimicrobial penetration by interacting with EPS and compromising biofilm integrity. Furthermore, HG1MB1’s ability to enhance MB diffusion into deeper biofilm layers aligns with existing evidence suggesting that hydrogel carriers can overcome biofilm resistance mechanisms by ensuring a more uniform drug distribution.^15^

In addition to the improved dye uptake, the elevated intracellular ROS levels generated by HG1MB1 upon irradiation (red light, 630 nm) contributed significantly to a greater reduction in both, EPS and microbial viability. The authors believe that enhanced uptake, due to the hydrogel consistency, combined with ROS production is likely a key factor for improving the efficacy of aPDT. Particularly when biofilms are known for their robust resistance to antimicrobial treatments due to their dense matrix and limited diffusion of therapeutic agents.^16,17^ The ROS-induced degradation of the biofilm and microbial inactivation is consistent with previous studies that showed ROS could effectively disrupt biofilm integrity and promote biofilm dispersion, thereby enhancing the effectiveness of antimicrobial treatments.^18^ These results are consistent with findings from a previous study,^7^ which demonstrated that the HG1MB1 formulation induces elevated levels of reactive oxygen species (ROS) across various hydrophilic environments. This supports the reproducibility and robustness of HG1MB1’s oxidative activity under different aqueous conditions.

In addition, HG1MB1 demonstrated a broad-spectrum activity against various microbial pathogens, including both bacteria and fungi. This makes it a versatile tool for treating biofilm-associated infections caused by a range of microorganisms. Research by O’Toole (2011)^19^ supports the applicability of hydrogels in targeting both bacterial and fungal biofilms, highlighting their potential as effective treatments for diverse pathogens. This broad-spectrum efficacy is an important feature of HG1MB1, as it can target a wide variety of biofilm-forming organisms, such as *S. aureus, P. aeruginosa*, and *C. albicans*.

Importantly, while MB uptake and ROS levels are elevated in the biofilm, their values in fibroblasts remain low, when treated with HG1MB1, minimizing the risk of cytotoxicity. This suggests that our hydrogel formulation selectively targets microbial biofilms without significantly affecting host cells. These findings align with Zhao et al.,^20^ who demonstrated that microbial cells are more susceptible to oxidative stress due to weaker antioxidant defenses compared to mammalian cells, and is further supported by studies on photoactive hydrogels, which selectively target biofilms while minimizing cytotoxicity to surrounding tissues, highlighting HG1MB1 as a promising approach for treating chronic biofilm-associated infections.^21,22^

Moreover, the lipid peroxidation analysis revealed that HG1MB1 was more effective than MB1 in inducing oxidative damage in biofilms following aPDT, aligning with the observed protein leakage results. Among the tested microorganisms, *S. aureus* exhibited the highest lipid peroxidation levels, followed by *P. aeruginosa* and *C. albicans*, suggesting varying susceptibilities to oxidative stress. However, these differences were not statistically significant, It can be inferred that these variations might be due to differences in cell wall composition and antioxidant defense mechanisms. *S. aureus* may retain more photosensitizer molecules, leading to increased ROS production and greater membrane disruption.

A study showed that oxidative stress induced by aPDT caused significant lipid peroxidation in bacterial biofilms, with *S. aureus* showing the highest susceptibility among the microorganisms tested.^23^ Similarly, was reported the structural differences in the cell wall composition of *S. aureus* contributed to its higher retention of photosensitizer molecules, enhancing ROS production and oxidative damage to the biofilm.^24,25^ Additionally, Zhang et al. (2021)^26^ investigated hydrogel-based systems loaded with photosensitizers, which were shown to increase oxidative stress within biofilms, leading to significant protein leakage and lipid peroxidation. Furthermore, Liu et al. (2022)^27^ reported that hydrogel matrices influenced the release rate of photosensitizers, which directly impacted the extent of oxidative stress, protein leakage, and lipid peroxidation within the biofilm.

Compared to biofilms, fibroblast cells displayed significantly lower lipid peroxidation levels, remaining within nonlethal and noncytotoxic thresholds. This supports the previous results, indicating that aPDT with HG1MB1 selectively induces oxidative stress in microbial biofilms while minimizing damage to host cells.

It is important to emphasize that HG1MB1 did not change the aPDT synergetic properties, once to achieve optimal biofilm inhibition with minimal damage to fibroblasts it is required a synergistic system, where MB is incorporated into the hydrogel will promote biofilm inhibition only upon exposure to a specific light source. This means that neither the hydrogel alone nor the light alone has an effect on microbial biofilms.

Thus, HG1MB1, represents a promising and effective strategy for treating chronic biofilm-associated infections. The unique properties of HG1MB1, such as its ability to enhance MB uptake and facilitate deeper penetration into biofilm matrices, significantly improve the efficacy of aPDT. By generating ROS upon light activation, HG1MB1 effectively disrupts biofilm integrity and inactivates microbial cells, demonstrating broad-spectrum activity against both bacterial and fungal pathogens. Importantly, the formulation selectively targets microbial biofilms without causing substantial damage to surrounding fibroblast cells, minimizing cytotoxicity. This selective action is further supported by fluorescence microscopy images, which reveal intact, viable fibroblasts alongside a significant reduction in biofilm formation.

Additionally, hydrogel as a gel-like offers several advantages over traditional hydrogel pads when it comes to treating biofilm. The increased fluidity allows the gel to more effectively conform to complex wound shapes or irregular surfaces, ensuring better coverage and deeper penetration into the biofilm. This enhanced contact improves the hydrogel’s ability to break down and displace the biofilm, which is often more resistant to treatment due to its protective structure. Furthermore, the liquid nature can facilitate the continuous exchange of moisture, creating an optimal environment for wound healing and maintaining hydration, which is critical for the removal of biofilm. In contrast, hydrogel pads may limit this interaction, potentially reducing their effectiveness in treating stubborn or extensive biofilm formations. It is important to highlight that, in our study, the hydrogel enhanced the effect of MB primarily by facilitating its delivery, as HG1 alone did not significantly inhibit biofilm formation. This suggests that the main role of HG1 was to transport MB to the deeper layers of the biofilm, where it exerted its inhibitory effect, ultimately reducing both the biofilm cell viability and overall biomass.

Thus, these findings highlight the potential of HG1MB1 as a valuable therapeutic tool for overcoming the challenges posed by chronic infections, offering a targeted, efficient, and safer alternative to conventional treatments.

## Experimental Section

### Bacterial and Fungal Strains and Culture Conditions

The microorganisms used in this study were *Staphylococcus aureus* (NCTC 11963), *Pseudomonas aeruginosa* (DSM 21482), and *Candida albicans* (ATCC 10231). The bacterial strains were routinely grown overnight in Brain Heart Infusion (BHI) broth under aerobic conditions at 37 °C in a shaker incubator (150 rpm). *Candida albicans* was cultured in Yeast Peptone Dextrose (YPD) broth at 30 °C – 150 rpm under aerobic conditions. A standard suspension of microbial cultures containing 1.0 × 10^7^ cells/mL was used for all experiments.

### Fibroblast Culture Conditions

Human dermal fibroblasts, adult (HDFa) were purchased from Gibco, Thermo Fisher Scientific. Cells were cultured in Dulbecco’s modified Eagle medium/Nutrient Mixture F-12 (DMEM/F12) (Gibco), supplemented with 10% fetal bovine serum (FBS) (Gibco) and 1% penicillin/streptomycin (Gibco). Cells were grown at 37̊C in a humidified atmosphere with 5% CO_2_. For the experiments, cells were seeded at 40,000 cells/ml density.

### MB-Based Photoactive Hydrogel Syntheses and Characterization

Briefly, the photoactive hydrogel used in this study was synthesized by mixing 5% pork gelatin with deionized water, followed by the addition of methylene blue at 1.0 mg/mL. Glutaraldehyde (GA) 25% was then added at a volume of 20 μL/mL as a cross-linking agent. A detailed methodology for the synthesis and characterization of the MB-based photoactive hydrogel is provided in a recently published study by the authors.^7^ It is important to highlight that the MB-based photoactive hydrogel will be referred to as HG1MB1, as cited in the previous article.

### Biofilm Formation

The biofilm formation was performed on a 12-well microtiter plate containing initially 1 mL of suspension cell (1 × 10^7^ cells/mL). The plates were incubated at 37 °C for 90 min without shaking to allow cell attachment to the well surface, simulating the reversible adhesion phase of biofilm formation. After the incubation, the wells were washed twice with 1 mL of sterile phosphate-buffered saline (PBS) to remove nonadherent cells. Subsequently, 2 mL of sterile BHI broth was added to the wells containing bacteria, while YPD broth was added to the wells with fungi. The plates were then maintained at 37 °C under shaking conditions (50 rpm) for 48 h to allow the development of mature biofilms.

### Uptake of Methylene Blue

#### Microbial Biofilm

The uptake of methylene blue by biofilms of *P. aeruginosa*, *S. aureus*, and *C. albicans* was evaluated using a modified version of the previously described by Shrestha and Kishen, 2012.^28^ After the formation of mature biofilms (described above), the biofilms were exposed to 2 mL of HG1MB1 and MB water-solution (1 mg/mL) under shaking conditions (50 rpm) at 37 °C for 30 min. The biofilm cultures were then harvested and washed with sterile PBS to remove any unbound dye and HG1MB1.

The biofilms were carefully scraped from the polystyrene wells and resuspended in 1 mL of sterile PBS to recover the biofilm-MB and biofilm-HG1MB1. After, they were centrifuged at 10,000 rpm for 10 min to allow the cells in the biofilm matrix to sediment along with the bound dye. The collected pellets were subjected to methanol extraction (1 mL) at room temperature for 1 h to release the cell-bound MB. After extraction, the samples were centrifuged again (10,000 rpm for 10 min) to separate the extracted dye from the biofilm matrix. The amount of MB uptake by the biofilm was quantified by measuring the absorbance of the extracted solution at 660 nm.

#### Fibroblast Cells

Approximately 10^5^ cells mL were seeded in 12-well microtiter plate containing DMEM/F12 medium. After 24 h the medium was changed to 2 mL of HG1MB1 and MB water-solution (1 mg/mL). The cells were incubated for 30 min at 37 °C in a dark humid atmosphere containing 5% CO_2_. At the end of the incubation period, the hydrogel and MB were removed, and the cell monolayer was carefully washed three times with PBS. One milliliter of trypsin (0.25% w/v)/EDTA (1 mM) was added to detach the cells from the wells, and the suspension cell was transferred to a conical tube, which was centrifuged for 1 min at 1000 rpm. The supernatant was discarded, and 1.0 mL of 2% (w/v) SDS was added to the pellet. After 1 h of agitation at room temperature, the samples treated with SDS were subjected to absorbance measurement at 660 nm for quantification of MB uptake.^29^

For both biofilm and fibroblasts, the percentage of MB uptake was calculated using the formula:

$$\mathrm{Uptake percentage of MB}=\frac{\mathrm{Amount of MB in dissolved pellet}}{\mathrm{Total amount of MB in dissolved pellet}}\times 100\%$$

This method allows for evaluating the interaction between the MB-biofilm and MB-fibroblast, providing insight into the hydrogel’s ability to penetrate and bind with them compared to the MB-water solution.

### aPDT Procedure

To evaluate the effect of aPDT on each microbial biofilm 1 mL of HG1MB1 was added to the 24-wells plate where the mature biofilms were formed. Then the plates were incubated in the dark at 37 °C for 30 min. After the incubation period, the plates were washed twice with PBS to remove the MB not binding to the biofilm. Then, the samples were irradiated for 30 min by a LED light device with a predominant wavelength of 630 nm (manufactured by FTMC), with a tip diameter of 11 mm. The intensity of light delivered was 18 mW cm^–2^. Cell viability was determined by using the micro drop technique and the colony counting was performed after 24 h of incubation at 37 °C to calculate the Colony-forming unit (CFU). The biofilm biomass was also quantified using the crystal violet assay.^32^

The same aPDT conditions were applied to the fibroblast cells, however, its viability was performed using MTT-microculture tetrazolium assay, a method of assessing cellular response to aPDT.^30^

Control samples were applied for comparison with the effectiveness of the HG1MB1. These were labeled as follows Table 1.

### EPS Quantification Assay

Congo red staining is a method that binds to polysaccharides within the EPS matrix.^31^ Thus, was used to quantify the biofilm production of EPS before and after aPDT with HG1MB1, and its respective controls (Table 1). For this methodology, the biofilms were formed on a 96-wells plate and after aPDT procedure (described above) they were incubated with 100 μL of Congo red (0.1% w/v) for 30 min at room temperature. After, the dye excess was washed off and the bound EPS-Congo red was eluted using ethanol. The amount of EPS was then quantified by measuring the absorbance at 495 nm using a plate reader.

### Intracellular Measurement of ROS

The intracellular measurement of ROS in microbial biofilms and fibroblasts was effectively performed using the fluorescent dye 2,7-dichlorofluorescein diacetate (DCFH-DA).^32^ This nonfluorescent compound penetrates the cell membrane and hydrolyzes by intracellular esterases to form DCFH, which, upon oxidation by ROS, is converted into fluorescent dichlorofluorescein (DCF). Biofilms and fibroblasts were cultured on suitable surfaces and incubated for 30 min with HG1MB1. After preincubation, the cells were washed with sterile PBS to remove the unbound HG1MB1. The fibroblasts were detached using Trypsin/EDTA, while microorganisms were gently scraped from the surface. The resulting pellets were then resuspended in PBS, treated with 50 μM of the relevant compound, and incubated for 10 min. The excess DCFH-DA was subsequently removed by centrifugation. The treated cells were then irradiated for 30 min, while control samples were evaluated as well (Table 1). The DCF fluorescence was measured using a microplate reader, with excitation at 485 nm and emission at 530 nm.

### aPDT Mechanism of Action

#### Analysis of Cytoplasmic Protein Leakage

To analyze cytoplasmic protein leakage from both microbial biofilms and fibroblast cells following aPDT with HG1MB1, a method based on the measurement of extracellular protein concentrations was employed.^33^ After the aPDT treatment, the supernatants were collected from both biofilm samples and fibroblast cultures. The biofilms were gently scraped to release extracellular proteins, while fibroblast cell layers were lysed as described above with trypsin/EDTA. Both were saved to a conical tube and centrifugated at 10,000 rpm for 5 min and the its pellets were collected. The optical density of each supernatant was measured at 595 nm using the Bradford assay, and the amount of protein leaked from the cells after aPDT was determined by comparing the absorbance values of the samples with the cytoplasmic leakage index.

Important to highlight that positive control samples were prepared by incubating bacterial cultures with cetyltrimethylammonium bromide (CTAB, 10 μg/mL) to establish the cytoplasmic leakage index for comparison with the protein leakage in test samples. The analyses were also conducted on the corresponding controls as outlined in Table 1.

#### Lipid Peroxidation Assay

Lipid peroxidation was assessed using the thiobarbituric acid reactive substances (TBARS) assay following aPDT with HG1MB1 on both microbial biofilms and fibroblast cells.^34^ Post-treatment, each sample was mixed with 10% ice-cold trichloroacetic acid (TCA) in a conic tube, followed by centrifugation at 14,000 rpm for 15 s to collect the homogenate. To this, 1 mL of 0.6% thiobarbituric acid (TBA) was added, and the mixture was boiled at 95 °C for 10 min to facilitate the formation of malondialdehyde (MDA), a product of lipid peroxidation. After cooling to room temperature, the reaction was centrifuged to remove any insoluble debris. The supernatant was then analyzed spectrophotometrically at 532 nm to measure the MDA-TBA adducts, and the results were quantified as nanomoles of MDA per milligram of protein. This assay provided a quantitative assessment of lipid peroxidation induced by aPDT, offering valuable insights into oxidative damage and membrane integrity in both microbial and mammalian cells.

This analysis was also performed on the controls described in Table 1.

### Analysis of Biofilms and Fibroblasts by Fluorescence Microscopy before and after aPDT with HG1MB1

Biofilms were cultivated in 24-well plates and assessed using the MycoLight bacterial viability assay kit. Following the removal of culture media, biofilms were gently washed three times with PBS to eliminate planktonic cells. After the aPDT treatment with HG1MB1, 100 μL of the prepared working dye solution was added to each well, and the plates were incubated at room temperature for 25 min in darkness to allow for staining. Unbound dye was removed by washing the wells twice with PBS.

Stained biofilms were visualized using a Nikon Eclipse Ti2 confocal laser scanning microscope equipped with a 20× objective lens. Fluorescence detection was performed at an excitation wavelength of 488 nm, with emission filters set to 510–530 nm for MycoLight Green (indicating live cells) and 600–660 nm for propidium iodide (indicating dead cells). Images were acquired using an Andor Zyla sCMOS camera integrated with the DSD2 differential spinning disc system, ensuring high-resolution fluorescence detection. To generate three-dimensional reconstructions of the biofilm architecture, Z-stack images were collected at 1 μm intervals. Data acquisition was conducted using Nikon Elements software, and subsequent image processing and analysis were performed with ImageJ (Fiji).

For fibroblast microscopy experiments, HDFa were seeded into 24-well plates and cultured for 48 h. Cells were then treated with HG1MB1 diluted in growth medium at a 1:1 ratio. After 30 min of incubation, cells were washed three times with PBS, and a fresh complete growth medium was added. Cells were subsequently irradiated with 630 nm light for 30 min, stained with 12.5 μg/mL propidium iodide (Roth), and analyzed using the EVOS FL Auto Onstage Incubator (Life Technologies).

### Statistical Analysis

Values are given as means and standard deviation of four separate experiments. Differences between HG1MB1 and its controls (Table 1) were tested for significance by one way ANOVA with Tukey posthoc test, mainly for antimicrobial analysis. *p*-values of less than 0.05 were considered significant.
